# Acid‐base imbalances and the association of blood‐gas variables, electrolytes, and biochemical analytes with outcome in hospitalized calves undergoing abdominal surgery

**DOI:** 10.1111/jvim.16618

**Published:** 2023-01-20

**Authors:** Florian M. Trefz, Corinna K. Lausch, Anna Rieger, Stine Giertzuch, Annette Lorch, Peter D. Constable

**Affiliations:** ^1^ Clinic for Ruminants with Ambulatory and Herd Health Services at the Centre for Clinical Veterinary Medicine LMU Munich Oberschleißheim Germany; ^2^ Department of Veterinary Clinical Medicine, College of Veterinary Medicine University of Illinois Urbana‐Champaign Urbana Illinois USA

**Keywords:** acidemia, gastrointestinal ileus, peritonitis, prognosis, venous oxygen tension

## Abstract

**Background:**

Surgical abdominal emergencies in calves are associated with a guarded prognosis and have the potential for complex metabolic derangements including acid‐base imbalances.

**Objectives:**

To perform a comprehensive analysis of acid‐base status and to assess the prognostic relevance of preoperative clinicopathologic variables in calves undergoing abdominal surgery.

**Animals:**

Hospital‐based study samples of 535 (dataset 1; DS1) and 83 calves (dataset 2; DS2).

**Methods:**

Retrospective (DS1) and prospective (DS2) case series.

**Results:**

In DS1, acidemia (pH <7.33) was present in 49.9%, whereas alkalemia (pH >7.37) was present in 30.7% of calves. Plasma L‐lactate, chloride, and serum inorganic phosphorus concentration accounted for 51.9%, 11.6% and 9.4% of the variation of venous blood pH, respectively. Classification tree analysis indicated that a negative outcome (death or euthanasia during hospitalization) was associated with venous pO_2_ ≤33.6 mm Hg, anion gap >18.3 and >22.9 mEq/L, serum albumin concentration ≤36.5 and ≤29.4 g/L, serum urea concentration >4.4 mmol/L, and plasma ionized calcium concentration ≤1.26 mmol/L. The area under the receiver operating characteristic curve of this model was 0.85 (95% CI: 0.82‐0.89, *P* < .001) and the resulting sensitivity and specificity for the prediction of nonsurvival at the optimal probability cut‐point of 0.5 was 89.8% and 65.7%, respectively. In DS2 the model had a similar sensitivity and specificity of 90.5% and 70%, respectively.

**Conclusions and Clinical Importance:**

Clinicopathologic imbalances and associated changes of acid‐base status are common in calves with surgical abdominal emergencies and have clinical utility for the prediction of a negative postoperative outcome.

## 1 INTRODUCTION

A variety of diseases including abomasal disorders, gastrointestinal ileus, or peritonitis can result in the clinical picture of an acute abdominal emergency in calves. Acute abdominal emergencies in calves frequently require surgical intervention, but they are associated with a reserved prognosis with reported case fatality rates ranging from 49% to 76%.^1^, ^2^, ^3^, ^4^, ^5^ This outcome has been attributed to irreversible organ damage because of a rapid disease progression, concomitant complications such as sepsis and endotoxemia, as well as a high prevalence of peritonitis and congenital malformations.^1^, ^2^, ^3^, ^4^, ^6^ For these reasons, there is interest in identifying biochemical preoperative prognostic factors that can be used to prediction of the outcome of therapy in affected animals in addition to clinical or ultrasonographic findings.

Acute abdominal emergencies in calves have the potential for complex metabolic derangements including marked hyper‐L‐lactatemia because of local or systemic hypoperfusion and decreased oxygen delivery, electrolyte imbalances, hemoconcentration, azotemia, as well as plasma protein alterations because of increased vascular permeability, leakage of albumin into the abdominal cavity, and the acute phase response to inflammation.^3^, ^4^, ^5^, ^7^, ^8^ These clinicopathologic changes potentially reflect disease severity, and they are associated with acid‐base derangements that can be demonstrated by application of the strong ion difference approach.^9^, ^10^, ^11^ For these reasons, acid‐base and related physicochemical variables might have clinical utility for predicting the outcome in critically‐ill calves undergoing abdominal surgery.

Little is known about acid‐base derangements and their prognostic utility in calves with surgical abdominal emergencies. The aim of the present study was therefore to perform a comprehensive analysis of acid‐base status, to determine the association between acid‐base variables and results of clinical biochemistry and electrolyte analysis, and to assess the prognostic relevance of theses variables in calves with a broad range of surgical abdominal emergencies.

## 2 MATERIAL AND METHODS

The primary analysis used a subset of calves in a dataset (dataset 1; DS1) that had been used in 2 retrospective studies focusing on the prognostic relevance of preoperative plasma L‐lactate and glucose concentrations.^4^, ^12^ Data from a subsequent prospective investigation (dataset 2; DS2) was analyzed to validate the predictive utility of a developed prognosis model from DS1 in terms of short‐ and midterm outcome to treatment.^3^

### 2.1 Calves

For DS1, the medical records of 587 calves with acute abdominal emergencies up to 6 months of age admitted to the Clinic for Ruminants, LMU Munich, between May 2005 and August 2015 were analyzed. Medical records were identified using log‐books and the clinic's hospital record database. Calves were selected for inclusion in this study if blood gas and electrolyte analysis and clinical biochemical analysis were performed on admission and abdominal surgery was performed during the first hours of hospitalization for reasons of an acute abdominal emergency. Furthermore, calves were also eligible for inclusion if they experienced an acute abdominal emergency during hospitalization and laboratory analysis was performed during the clinical diagnostic procedure before initiation of surgery. A decision toward surgical intervention was made on the basis of typical clinical signs including signs of abdominal pain (colic), abdominal distension, positive right‐sided percussion and succession auscultations, tensed abdominal wall, dehydration or the absence of feces on digital palpation per rectum. Furthermore, the clinical diagnostic procedure included an ultrasonographic examination if deemed necessary by the responsible clinician.

The validation dataset, DS2, consisted of 83 calves up to 7 months of age admitted to the Clinic for Ruminants with Ambulatory and Herd Health Services, LMU Munich, between August 2015 and December 2016. Calves were prospectively enrolled if they were suffering from an acute abdominal emergency that required surgical intervention.

### 2.2 Perioperative treatment and intraoperative diagnoses

Details concerning the clinical examination, surgical procedures, and treatment of calves can be found elsewhere.^3^, ^4^ In both study samples, categorization was assigned based on the anatomical location of the underlying problem (abomasum, small intestine, large intestine, multiple locations in the gastrointestinal tract, or abdominal cavity) as well as by the primary clinical diagnosis. The latter was determined based on the documented clinical and intraoperative findings and was considered to be the single most detrimental diagnosis that was responsible for the clinical picture and condition of the calf, even if other contributing problems were present.

### 2.3 Outcome of therapy

A positive outcome of therapy (survival; categorized as PO) in DS1 was defined as discharge from the hospital. Calves with a negative outcome (nonsurvival; categorized as NO) died or were euthanized during hospitalization because of fatal intraoperative findings, severe deterioration in general condition, ongoing signs of gastrointestinal ileus, or animal welfare reasons.

Two outcome definitions were used for the prospective study sample (DS2). The outcome of calves was evaluated until hospital discharge and additionally after 3 months after discharge by means of a telephone call to the farmer as described.^3^ In brief, calves in DS2 were categorized as PO if discharged calves were still in the herd, grew at a similar rate to at least 75% of their herdmates, had a satisfied owner, or were sold without discount. In contrast, calves were categorized as NO if they died or were euthanized during hospitalization or if discharged animals were reported to show a slow growth rate, had an unsatisfied owner, were sold at a discount, or had died within the 3‐month period after discharge from the hospital.

### 2.4 Laboratory analyses

Blood samples were collected from the jugular vein on admission to the hospital or in a preoperative situation before any treatment was administered. Lithium‐heparinized blood samples were anaerobically collected using a 2‐mL polypropylene syringe and blood pH, pCO_2_, and plasma sodium, chloride, potassium, and ionized calcium concentrations determined by means of blood pH, gas, and electrolyte analyzers using ion selective electrodes (Rapidlab 865 [from 2005 to 2012] and Rapidpoint 405 [from 2012 to 2016] analyzers, Siemens Healthcare Diagnostics Inc, Tarrytown). Blood pH and pCO_2_ were corrected for rectal temperature using the same equation for both analyzers.

Automatic analyzers were used for additional serum and plasma biochemical analysis (Hitachi 911 [from 2005 to 2012] and Cobas c311 [from 2012 to 2015] analyzers, Roche Diagnostics, Mannheim, Germany). Serum samples (plain tubes) were assayed for concentrations of urea (urease), creatinine (picric acid), total protein (biuret), albumin (bromcresol green), and inorganic phosphorus (molybdenum). Concentrations of L‐lactate were determined from blood samples containing lithium heparin and potassium fluoride as a glycostatic agent by means of a lactate oxidase assay.

### 2.5 Calculations

Actual bicarbonate concentration (*c*HCO_3_^−^) and base excess of blood (abbreviated as BE [B], also called in vitro base excess) was automatically calculated by the blood gas analyzers as described.^9^, ^13^

An estimate of the unmeasured anion concentration was obtained by calculating the anion gap (AG) in mEq/L, whereby:

(1) $$\mathrm{AG}=\left(c{\mathrm{Na}}^{+}+cK^{+}\right)-\left(c{\mathrm{Cl}}^{-}+c{{\mathrm{HCO}}_{3}}^{-}\right).$$

In addition to the traditional Henderson‐Hasselbalch acid‐base model, the simplified quantitative physiochemical strong ion approach^14^ was used in order to allow a more comprehensive assessment of the acid‐base status of calves. The measured strong ion difference obtained from 5 strong ions (SID_5_; mEq/L) was calculated using the measured value for [Ca^2+^] determined by ion‐selective potentiometry and assigning a charge of −1 to L‐lactate assuming 100% dissociation such that:

(2) $${\mathrm{SID}}_{5}=c{\mathrm{Na}}^{+}+cK^{+}+c{\mathrm{Ca}}^{2+}-c{\mathrm{Cl}}^{-}-c\left(L-\text{lactate}\right).$$

In order to be able to assess the relative importance of sodium and chloride imbalances on acid‐base status, the strong ion difference calculated from the plasma concentrations of sodium, potassium and chloride (SID_3_; mEq/L) was additionally obtained by^15^:

(3) $${\mathrm{SID}}_{3}=c{\mathrm{Na}}^{+}+cK^{+}-c{\mathrm{Cl}}^{-}.$$

The concentration of nonvolatile weak acids (*A*_tot_) in mmol/L was calculated from plasma concentrations of total protein^15^:

(4) $$A_{\mathrm{tot}}=0.343\times c\text{total protein}.$$

An estimate of the unmeasured strong ion concentration was obtained by calculating the strong ion gap (SIG) in mEq/L, defined as the difference between the plasma concentration of unmeasured strong cations and unmeasured strong anions. This was performed by using the experimentally determined value for *A*_tot_, the experimentally determined value for the negative logarithm of dissociation constant of plasma nonvolatile weak acids (pK_a_ = 7.08), and the following equation^15^:

(5) $$\mathrm{SIG}=\left[A_{\mathrm{tot}}/\left(1+10^{\left(7.08-\mathrm{pH}\right)}\right)\right]-\mathrm{AG}.$$

The first expression on the right‐hand side of the SIG equation represents the net negative charge in mEq/L of a lumped group of nonvolatile weak acids (*A* ^−^) in plasma, consisting of albumin, globulins, and dihydrogen phosphate. Calculated values for SIG were also corrected for the measured concentrations of L‐lactate and Ca^2+^ to obtain the concentration of still unidentified strong ions (USI) in mEq/L for the calculation of the effective strong ion difference (SID_eff_) such that:

(6) $$\mathrm{USI}=\mathrm{SIG}+c\left(L-\text{lactate}\right)-c{\mathrm{Ca}}^{2+},$$

(7) $${\mathrm{SID}}_{\mathrm{eff}}={\mathrm{SID}}_{5}+\mathrm{USI}={\mathrm{SID}}_{3}+\mathrm{SIG}.$$

In order to detect pH‐dependent effects on ionized calcium concentrations, measured Ca^2+^ was also corrected for change in pH from 7.4 by use of the following equation^16^:

(8) $$c{{\mathrm{Ca}}^{2+}}_{7.4}=c{\mathrm{Ca}}^{2+}\times 10^{-0.23\times \left(7.4-\mathrm{pH}\right)}.$$

### 2.6 Statistical analysis—DS1

Statistical analyses were performed using SPSS for windows (version 26.0, IBM Corp., Armonk, New York), GraphPad Prism (version 7.01, GraphPad software) and the partykit package (version 1.1‐1) in R (version 3.4.4, R Core Team). Values of *P* < .05 were considered statistically significant. Because most of the data were not normally distributed, as indicated by the Shapiro‐Wilk test and visual examinations of QQ plots, data are presented as medians and interquartile ranges (*Q*_1_/*Q*_3_).

Spearman's correlation coefficient (*r*_*s*_) was calculated to characterize associations between variables. Stepwise forward multivariable regression models were additionally constructed, including measured variables of clinical pathology that were significantly correlated with the dependent variables or considered relevant from a biological standpoint of view. To minimize the effects of collinearity and ensure an appropriately low variance inflation factor for individual values, when 2 variables were closely correlated with each other (*r*_*s*_ > 0.7), only the variable that had the highest *r*_*s*_ to the dependent variable was entered into the model.^9^ The relative importance of the included variables was assessed by the order of entry into the model as well as by the change in the model *R* ^2^ value (Δ*R* ^2^). Standardized residual plots of each multivariable model were examined to confirm an approximately normal distribution of residuals. The models were calculated with raw and log‐transformed data; the analysis on raw data is presented because the log‐transformed data did not yield a better model fit. Interaction terms were not investigated for each significant predictor in the multivariable models.

Categorized variables were compared using a chi‐square test or Fisher's exact test if the expected frequency in 1 or more of the cells was <5. Comparisons of continuous variables between the 5 defined anatomical location groups of calves based on intraoperative lesion localization were assessed using a Kruskal‐Wallis test. For the subsequent pair‐wise comparisons the Mann‐Whitney *U*‐test was used and the level of significance adjusted using the Bonferroni method (total of 10 comparisons, providing an experiment‐wise comparison *P* < .005). Mann‐Whitney *U*‐tests were also used for the comparison of laboratory variables between calves with a PO and NO. Survival rates in relation to deciles of selected variables were also evaluated by comparing survival rates of each decile to the survival rate of calves of the decile that best lay within the reference range.^13^ If 2 decile groups were equally eligible as a reference group, that decile group with the lower survival rate was used in order to avoid a potential selection bias. For this analysis, the level of significance was also adjusted using the Bonferroni method (total of 9 comparisons, providing an experiment‐wise comparison *P* < .006). For multivariable modeling of prognostic factors, data were analyzed using classification tree analysis to identify additional cut‐points as potential mortality predictors for subsequent evaluation using binary logistic regression analysis.^4^, ^12^, ^13^ Because of our previous finding that survival rates of affected calves <3 weeks of age are considerably low,^4^, ^12^ age was additionally included in this analysis. Variables identified as statistically significant predictors during classification tree analysis were subsequently entered as categorized outcome variables into multivariable regression models using a stepwise backward procedure with Wald *P* < .05 as selection criterion. The fit of the final model was evaluated by means of the Hosmer‐Lemeshow goodness‐of‐fit test. Furthermore, the predictive ability of the model was assessed by calculating the area under the receiver‐operating characteristic (ROC) curve and the sensitivity, specificity, positive predictive value (PPV), and negative predictive value (NPV) at the optimal cut‐point identified on the basis of the Youden index.

### 2.7 Statistical analysis—DS2

The established model from DS1 was validated in terms of its predictive utility until hospital discharge and 3 months later using data from DS2.

## 3 RESULTS

### 3.1 General conditions

Information on ionized plasma calcium concentration or plasma potassium concentration were not available for 10 calves and 1 calf, respectively. Also, information about serum inorganic phosphorus concentration was missing in 42 calves as it was not included in the routine biochemistry panel between January 2008 and January 2009. Data for these 3 analytes were assumed to be missing at random. Complete information for the laboratory variables of interest was available for 535 calves and this dataset was therefore used in the analysis reported here. Most of the calves were German Fleckvieh (*n* = 476; 89%), which is the most common dairy breed in Bavaria. The median age (*Q*_1_/*Q*_3_) was 4.9 (1.7/8.7) weeks.

### 3.2 Diagnoses and outcome of therapy

Individual diagnoses of calves of DS1 are listed in Table 1. A total of 89 calves had an abomasal disorder (16.6%), 120 calves (22.4%) had a disorder located in the small intestine and 81 calves (15.1%) had a disorder in the large intestine. Multiple parts of the gastrointestinal tract were affected in 112 calves (20.9%), and 133 calves (24.9%) had a diagnosis of peritonitis. A total of 363 calves (67.9%) did not survive until hospital discharge of which 303 calves were euthanized and 60 calves died spontaneously. Survival rates between the 5 defined anatomical location groups of calves differed (*P* < .001) and were 57.3% for calves with an abomasal disorder, 37.5% for calves with a small intestinal disorder, 18.5% for calves with a large intestinal disorder, 50% for calves with a disorder of multiple parts of the gastrointestinal tract, and 3.8% for calves with a diagnosis of peritonitis, respectively.

**TABLE 1:** Individual diagnoses of calves of the retrospective study sample (*n* = 535)

Lesion location and individual diagnoses	Number of calves
Abomasum (16.6%)	
Right‐sided dilatation^a^	50
Volvulus^b^	34
Miscellaneous^c^	5
Small intestine (22.4%)	
Volvulus	44
Hernial incarceration	19
Impaction with feed components	19
Strangulation^d^	18
Intussusception	8
Malformations	7
Dislocation	5
Large intestine (15.1%)	
Malformations	35
Intussusception	15
Cecal torsion or colonic volvulus	16
Cecal dilatation	10
Impaction with feed components	5
Multiple locations (20.9%)	
Mesenteric volvulus	70
Bloat/gas colic^e^	32
Paralytic ileus	10
Abdominal cavity (24.9%)	
Peritonitis^f^	133

^a^ Defined as an enlargement and displacement of the abomasum attributable to gas or fluid with the pylorus located caudally, and the absence of any palpable twisting of the omentum on the axial surface of the abomasum.

^b^ Defined as an enlargement and displacement of the abomasum attributable to gas or fluid with the pylorus located cranially, and the presence of any palpable twisting of the omentum on the axial surface of the abomasum, and the requirement of a clockwise or anticlockwise rotation of the abomasum during surgical correction when viewed from the right side of the abdomen.

^c^ Including 2 calves with abomasal impaction and 3 calves with an incarceration in an umbilical hernia.

^d^ Intestinal strangulations through intra‐abdominal adhesions, navel structures, or mesenteric defects.

^e^ Characterized by gaseous distension of multiple parts of the gastrointestinal tract.

^f^ Defined as visible inflammation of the serosal surfaces and peritoneal fluid, characterized by the presence of fibrin or hyperemia on serosal surfaces, or cloudy peritoneal fluid. Peritonitis was considered to be related to perforated abomasal ulcers (*n* = 33; of which 25 had an omental bursitis), intestinal contusion or perforation (*n* = 29), infection of intraabdominal umbilical structures (*n* = 26), necrosis of the intestinal wall (*n* = 21), septicemia (*n* = 21), rupture of the abomasum (*n* = 2), and wound dehiscence after previous laparotomy (*n* = 1).

In prospective DS2, a total of 15 calves (18.1%) suffered from an abomasal disorder, 14 calves (16.9%) had a disorder located in the small intestine and 13 calves (15.7%) had a disorder in the large intestine. Multiple parts of the gastrointestinal tract were affected in 21 calves (25.3%), and 20 calves (24.0%) had a diagnosis of peritonitis.

A total of 59 calves (71.1%) did not survive until hospital discharge, of which 53 calves were euthanized and 6 calves died spontaneously. Based on the follow‐up interviews, 4 out of 24 discharged calves were additionally allocated to the group of calves with NO. Therefore, the overall proportion of calves in DS2 with PO after 3 months was 24.1%. Similarly, as in DS1, the survival rates differed significantly (*P* = .008) between the 5 defined anatomical location groups of calves and were 40.0% for calves with an abomasal disorder, 38.1% for calves with a disorder of multiple parts of the gastrointestinal tract, 28.6% for calves with a small intestinal disorder, 15.4% for calves with a large intestinal disorder, and 0% for calves with a diagnosis of peritonitis after the 3‐month observation period, respectively.

### 3.3 Findings of acid‐base and clinicopathologic analyses in calves of DS1

The reference range for selected acid‐base and clinicopathologic variables of interest are presented in Table 2. Acidemia (pH <7.33) was present in 267 calves of DS1 (49.9%), whereas alkalemia (pH >7.37) was present in 164 calves (30.7%). The proportion of acidemic calves was significantly (*P* = .017) higher in calves aged <60 days (53.1%) than in older calves (41.5%). In contrast, the proportion of alkalemic calves was significantly higher (*P* = .002) in calves ≥60 days of age (40.8%) than in younger calves (26.8%).

**TABLE 2:** Median and interquartile ranges for acid‐base variables, electrolytes, and serum or plasma biochemical analytes in 535 calves with surgical abdominal emergencies categorized by intraoperative lesion localization

		Abomasum (n = 89)	Small intestine (n = 120)	Large intestine (n = 81)	Multiple locations (n = 112)	Peritonitis (n = 133)	
Variable	Reference range^15^, ^17^, ^18^	$\tilde{x\;}$ (*Q*_1_/*Q*_3_)	$\tilde{x\;}$ (*Q*_1_/*Q*_3_)	$\tilde{x\;}$ (*Q*_1_/*Q*_3_)	$\tilde{x\;}$ (*Q*_1_/*Q*_3_)	$\tilde{x\;}$ (*Q*_1_/*Q*_3_)	*P*‐value
Henderson‐Hasselbalch acid‐base model							
pH	[7.33 to 7.37]	7.34 (7.25/7.38)^a^	7.34 (7.27/7.39)^a^	7.35 (7.29/7.39)^a^	7.29 (7.13/7.36)^b^	7.33 (7.25/7.4)^a^	<.001
pCO_2_ (mm Hg)	[43.5 to 54]	55.4 (48.7/60.1)	51.5 (47.9/57.2)	52.3 (48.9/60.2)	54.0 (49.6/57.2)	54.8 (49.0/60.2)	.18
pO_2_ (mm Hg)	[36 to 46.5]	34.8 (28.9/40.8)^a^	34.4 (29.2/39.2)^a^	32.8 (27.7/37.8)^ab^	35.3 (30.6/41.5)^a^	29.5 (26.0/34.8)^b^	<.001
HCO_3_^−^ (mmol/L)	[23 to 29]	27.6 (23.2/32.5)^a^	26.5 (22.0/30.5)^a^	28.1 (24.9/31.4)^a^	23.4 (16.5/29.0)^b^	27.1 (21.6/32.2)^a^	<.001
BE(B) (mmol/L)	[−3.5 to 3.5]	1.8 (−4.0/6.3)^a^	1.0 (−5.0/4.6)^a^	2.1 (−1.8/5.2)^a^	−2.7 (−11.9/2.4)^b^	0.9 (−5.5/6.4)^a^	<.001
AG (mEq/L)	[8.9 to 15.0]	14.7 (11.8/21.2)^ac^	14.1 (10.3/22.9)^ab^	11.8 (7.1/17.8)^b^	17.5 (12.9/29.3)^c^	16.6 (11.6/23.6)^ac^	<.001
SID acid‐base model							
*A*_tot_ (mmol/L)	[15.9 to 21.2]	19.3 (17.7/21.8)^ab^	18.8 (17.5/20.8)^ab^	18.1 (16.3/20.4)^a^	19.7 (17.8/21.3)^b^	18.6 (15.7/21.2)^ab^	.006
A^−^ (mEq/L)	n.a.	12.3 (10.5/14.4)	11.9 (10.7/13.2)	11.6 (10.3/12.8)	11.8 (10.1/13.0)	11.9 (9.9/13.8)	.30
SID_3_ (mEq/L)	[38.3 to 47.7]	42.5 (39.5/46.9)^ac^	41.9 (37.0/46.8)^ab^	40.9 (36.9/43.7)^b^	42.8 (39.0/48.1)^c^	44.1 (39.8/51.5)^c^	<.001
SID_5_ (mEq/L)	n.a.	40.2 (36.1/44.0)^ab^	39.4 (35.6/43.2)^ab^	39.0 (35.3/42.5)^a^	38.0 (33.8/42.0)^a^	41.0 (36.1/46.9)^b^	.001
SID_eff_ (mEq/L)	[37.3 to 51.5]	39.8 (34.4/45.9)^ad^	38.6 (33.5/42.6)^abd^	40.0 (35.8/43.2)^cd^	35.9 (28.1/41.1)^b^	38.6 (32.8/46.1)^d^	<.001
USI (mEq/L)	[−2 to 0]	0.4 (−3.4/3.8)^ac^	−0.4 (−6.0/2.7)^abcd^	1.0 (−3.8/4.0)^cd^	−2.6 (−7.2/0.9)^b^	−1.8 (−6.5/2.1)^ab^	<.001
SIG (mEq/L)	[−3 to 3]	−3.0 (−8.5/1.4)^ac^	−1.8 (−9.7/2.6)^ac^	0.8 (−4.7/4.0)^a^	−5.6 (−19.1/−0.3)^bd^	−4.7 (−10.6/0.3)^cd^	<.001
Electrolytes							
Na^+^ (mmol/L)	[132 to 152]	136.1 (132.0/139.6)^ab^	134.6 (131.6/138.1)^a^	134.9 (131.8/138.6)^a^	137.4 (134.2/140.3)^b^	135.3 (132.2/139.5)^ab^	.002
K^+^ (mmol/L)	[3.9 to 5.8]	4.17 (3.76/4.58)^a^	4.35 (3.90/5.09)^ab^	4.38 (4.07/4.78)^ab^	4.44 (3.90/5.07)^b^	4.41 (3.89/5.16)^b^	.026
Cl^−^ (mmol/L)	[95 to 110]	97 (93/100)^ac^	97 (93/101)^abc^	99 (96/102)^b^	99 (95/102)^b^	96 (90/101)^c^	<.001
Ca^2+^ (mmol/L)	[1.0 to 1.3]	1.17 (1.08/1.24)^a^	1.16 (1.08/1.22)^a^	1.16 (1.13/1.23)^ab^	1.20 (1.13/1.28)^b^	1.12 (1.04/1.18)^c^	<.001
Ca^2+^_7.40_ (mmol/L)	[1.0 to 1.3]	1.09 (1.03/1.19)^a^	1.10 (1.04/1.16)^a^	1.13 (1.08/1.19)^a^	1.11 (1.05/1.19)^a^	1.05 (0.99/1.13)^b^	<.001
Biochemical analysis							
L‐lactate (mmol/L)	[≤2.2]	4.7 (2.6/9.0)^ac^	3.5 (2.1/6.4)^ab^	2.9 (1.7/5.0)^b^	5.9 (3.0/11.2)^c^	4.6 (2.9/7.3)^ac^	<.001
Total protein (g/L)	[59 to 70]	56.2 (51.6/63.5)^ab^	54.9 (50.9/60.7)^ab^	52.9 (47.4/59.4)^a^	57.5 (51.8/62.0)^b^	54.3 (45.7/61.8)^ab^	.006
Albumin (g/L)	[30 to 40]	32.8 (30.3/35.6)^a^	30.2 (27.9/33.7)^b^	28.5 (25.8/31.6)^b^	33.1 (30.1/36.5)^a^	27.4 (25.1/30.7)^b^	<.001
Globulin (g/L)	[30 to 40]	23.8 (19.6/28.9)	24.6 (20.9/30.7)	23.9 (20.4/28.8)	23.8 (20.0/28.4)	25.2 (20.1/32.0)	.29
A/G ratio	n.a.	1.41 (1.14/1.74)^a^	1.22 (1.02/1.48)^b^	1.21 (0.98/1.50)^b^	1.41 (1.12/1.66)^a^	1.09 (0.86/1.39)^b^	<.001
Phosphorus (mmol/L)	[2.0 to 3.5]	2.8 (2.5/3.4)^ac^	2.6 (2.2/3.8)^ab^	2.3 (2.1/3.1)^b^	3.1 (2.5/4.2)^c^	3.0 (2.2/4.2)^ac^	<.001
Urea (mmol/L)	[≤5.5]	6.4 (4.0/11.8)^a^	6.5 (4.6/13.4)^a^	6.1 (4.3/10.5)^a^	4.9 (3.0/6.6)^b^	10.2 (6.2/18.5)^c^	<.001
Creatinine (μmol/L)	[110 to 180]	130 (97/161)	124 (91/182)	114 (83/158)	129 (94/171)	136 (87/242)	.11

*Note*: Different letters indicate a statistical significant difference between groups (*P* < .005).

Abbreviations: $\tilde{x\;}$ (*Q*_1_/*Q*_3_), median and respective interquartile range; A/G, albumin‐to‐globulin‐ratio; AG, anion gap; *A*_tot_, concentration of nonvolatile weak acids; BE(B), base excess of blood (in vitro base excess); n.a., not available; pCO_2_, partial pressure of carbon dioxide; pO_2_, partial pressure of oxygen; SID_3_, strong ion difference calculated from 3 strong ions; SID_5_, strong ion difference calculated from 5 strong ions; SID_eff_, effective strong ion difference; SIG, strong ion gap; USI, unidentified strong ions.

Use of the Henderson‐Hasselbalch approach to evaluating acid‐base balance (by evaluating pCO_2_, *c*HCO_3_, and AG) indicated the presence of an acid‐base derangement in 450 calves (84.1%), of which 427 calves were suffering from a mixed acid‐base disorder. Specific findings are reported in Table 3. A hypochloremic metabolic alkalosis (*c*HCO_3_^−^ > 29 mmol/L; *c*Cl^−^ < 95 mmol/L) was diagnosed in 88 calves of which 19 calves had an abomasal disorder, 18 calves had a disorder in the small intestine, 8 calves had a large intestinal abnormality, 10 calves had abnormalities in multiple locations in the gastrointestinal tract, and 33 calves were diagnosed with peritonitis.

**TABLE 3:** Relative frequency of acid‐base disorders in calves of the retrospective study sample (*n* = 535)

Finding	Number of calves	Percentage of study sample
Henderson‐Hasselbalch acid‐base model		
Metabolic acidosis (HCO_3_^−^ < 23 mmol/L)	166	31.0%
Metabolic alkalosis (HCO_3_^−^ > 29 mmol/L)	185	34.6%
Venous pCO_2_ acidosis (pCO_2_ > 54 mm Hg)	252	47.1%
Venous pCO_2_ alkalosis (pCO_2_ < 43.5 mm Hg)	38	7.1%
Increased anion gap (>15 mEq/L)	273	51.0%
SID acid‐base model		
Strong ion (SID_3_) acidosis (SID_3_ < 38.3 mEq/L)	135	25.2%
Strong ion (SID_3_) alkalosis (SID_3_ > 47.7 mEq/L)	139	26.0%
Buffer ion acidosis (*A*_tot_ > 21.2 mmol/L)	129	24.1%
Buffer ion alkalosis (*A*_tot_ < 15.9 mmol/L)	76	14.2%
Increased strong ion gap (<−3 mEq/L)	273	51.0%

Use of the simplified strong ion approach to evaluating acid‐base balance (by evaluating pCO_2_, SID_3_, SIG, and *A*_tot_) indicated the presence of an acid‐base derangement in 499 calves (93.3%), of which 322 calves had a mixed acid‐base disorder. Specific findings are also reported in Table 3.

Median and interquartile ranges for variables of acid‐base status and results of clinical biochemistry and electrolyte analysis in the 5 defined groups of calves based on intraoperative lesion localization are reported in Table 2. Statistical significant differences were detected for the vast majority of evaluated variables. Calves with disorders of multiple parts of the gastrointestinal tract had lowest values for venous blood pH, bicarbonate concentration, BE(B), SID_eff_, and SIG and highest values for AG and plasma L‐lactate concentration when compared to other groups. Lowest values for serum albumin concentration, and highest values for serum urea and creatinine concentration were detected in the group of calves with a diagnosis of peritonitis.

### 3.4 Association between clinicopathologic findings and acid‐base variables in calves of DS1

**FIGURE 1:**
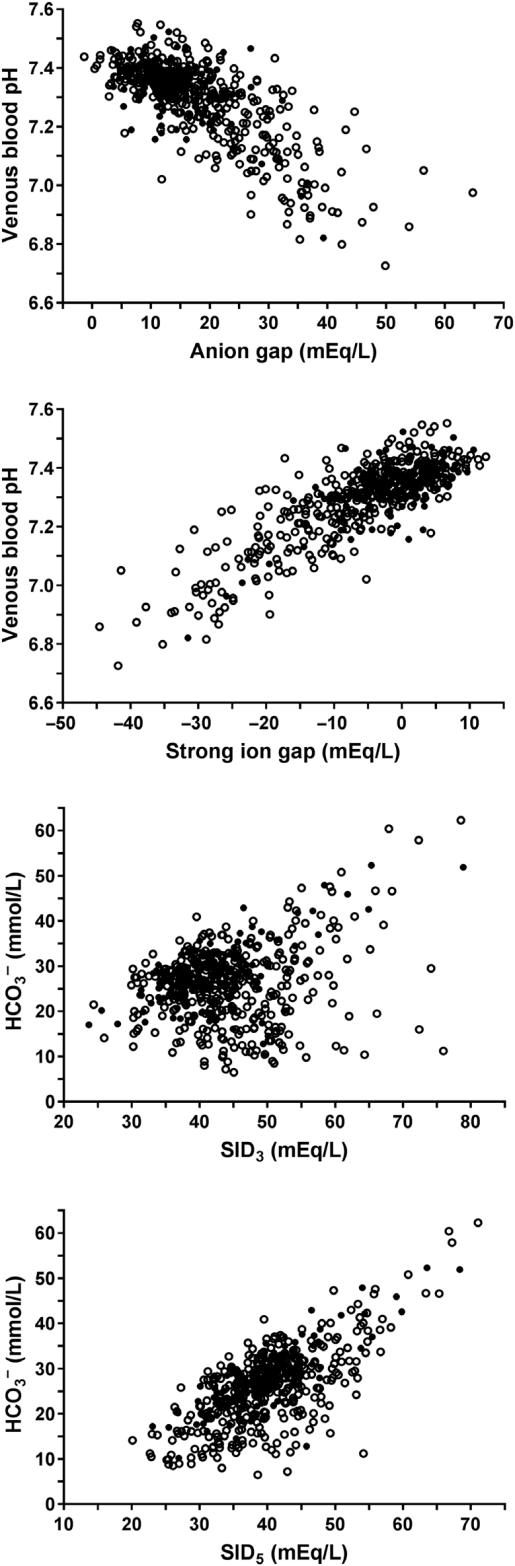
Scatterplots illustrating the association of venous blood pH with anion gap, strong ion gap, and plasma bicarbonate concentration with strong ion difference calculated from 3 (SID_3_) and 5 (SID_5_) strong ions in 535 hospitalized calves with surgical abdominal emergencies. Open circles indicate calves with a negative outcome of therapy (death or euthanasia) whereas closed circles indicate calves with a positive outcome

**FIGURE 2:**
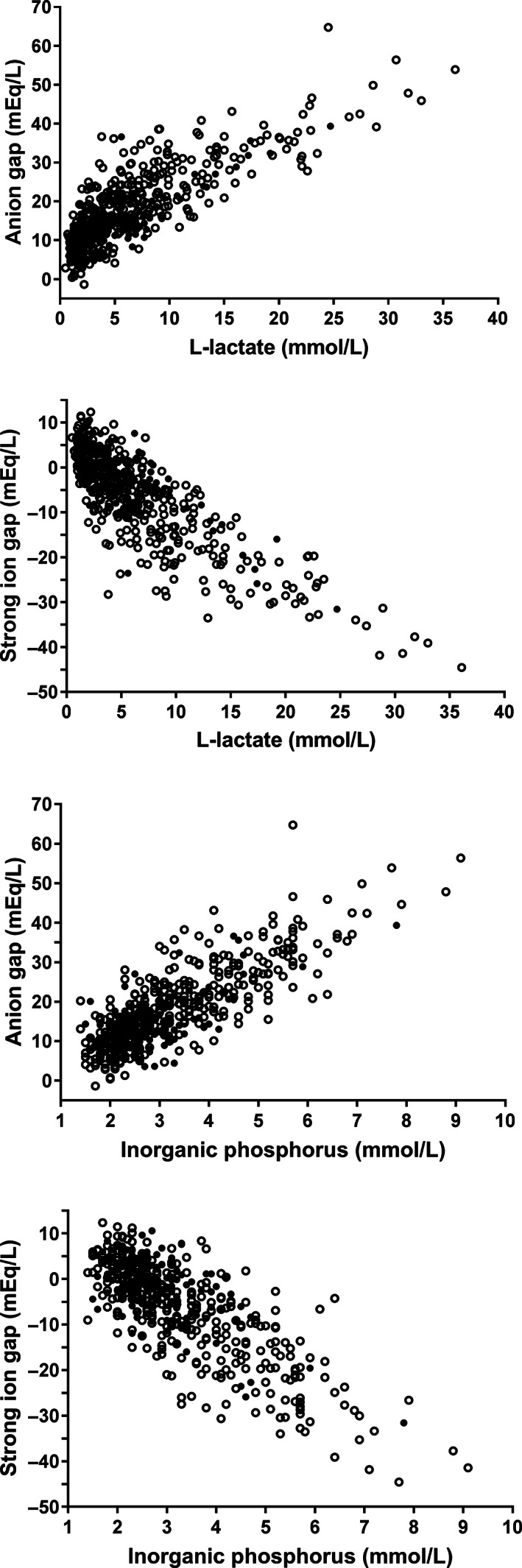
Scatterplots illustrating the association of anion gap and strong ion gap with plasma L‐lactate and serum inorganic phosphorus concentrations in 535 hospitalized calves with surgical abdominal emergencies. Open circles indicate calves with a negative outcome of therapy (death or euthanasia) whereas closed circles indicate calves with a positive outcome

Spearman's correlation coefficients between clinicopathologic factors and variables of acid‐base status of calves of DS1 can be found in Table S1. The quantified unmeasured anion (AG) and strong anion concentration (SIG) was closely correlated to venous blood pH (*r*_s_: −0.67 and 0.74), actual bicarbonate concentration (*r*_s_: −0.59 and 0.66), and BE(B) (*r*_s_: −0.64 and 0.71), respectively. Scatterplots illustrating the association of venous blood pH with AG and SIG as well as of plasma bicarbonate concentration with SID_3_ and SID_5_ are shown in Figure 1. In order to further investigate associations with venous blood pH, pCO_2_, as well as serum or plasma concentrations of L‐lactate, total protein, inorganic phosphorus, sodium, chloride, potassium, and creatinine were entered into an additional multivariable stepwise forward linear regression model. Plasma L‐lactate, chloride, and serum inorganic phosphorus accounted for 51.9%, 11.6% and 9.4% of the variation of venous blood pH, respectively (Table 4). Plasma L‐lactate and serum inorganic phosphorus concentrations were also closely correlated to AG and SIG, respectively (Figure 2).

**TABLE 4:** Results of a stepwise forward multivariable linear regression analysis for the prediction of venous blood pH in 535 calves with surgical abdominal emergencies

Order of entry	Variable^a^	∆*R* ^2^	Model *R* ^2^	Coefficient	±SE	*P*‐value	Variance inflation factor
	Constant	–	–	7.849	0.07	<.001	–
1	L‐lactate	0.519	0.519	−0.014	0.001	<.001	2.19
2	Chloride	0.116	0.635	−0.011	0.001	<.001	1.88
3	Phosphorus	0.094	0.729	−0.048	0.004	<.001	3.11
4	Sodium	0.042	0.77	0.007	0.001	<.001	1.88
5	pCO_2_	0.018	0.789	−0.002	<0.001	<.001	1.39
6	Potassium	0.003	0.792	−0.01	0.003	.003	1.48
7	Total protein	0.002	0.794	−0.001	<0.001	.022	1.28

Abbreviations: ∆*R* ^2^ = change of *R* ^2^ after adding of respective variable to the multivariable regression model; pCO_2_ = partial pressure of carbon dioxide.

^a^ Serum creatinine concentrations were eliminated at the *α* = 5% level.

### 3.5 Prognostic utility of preoperative acid‐base and biochemical variables in calves of DS1

**FIGURE 3:**
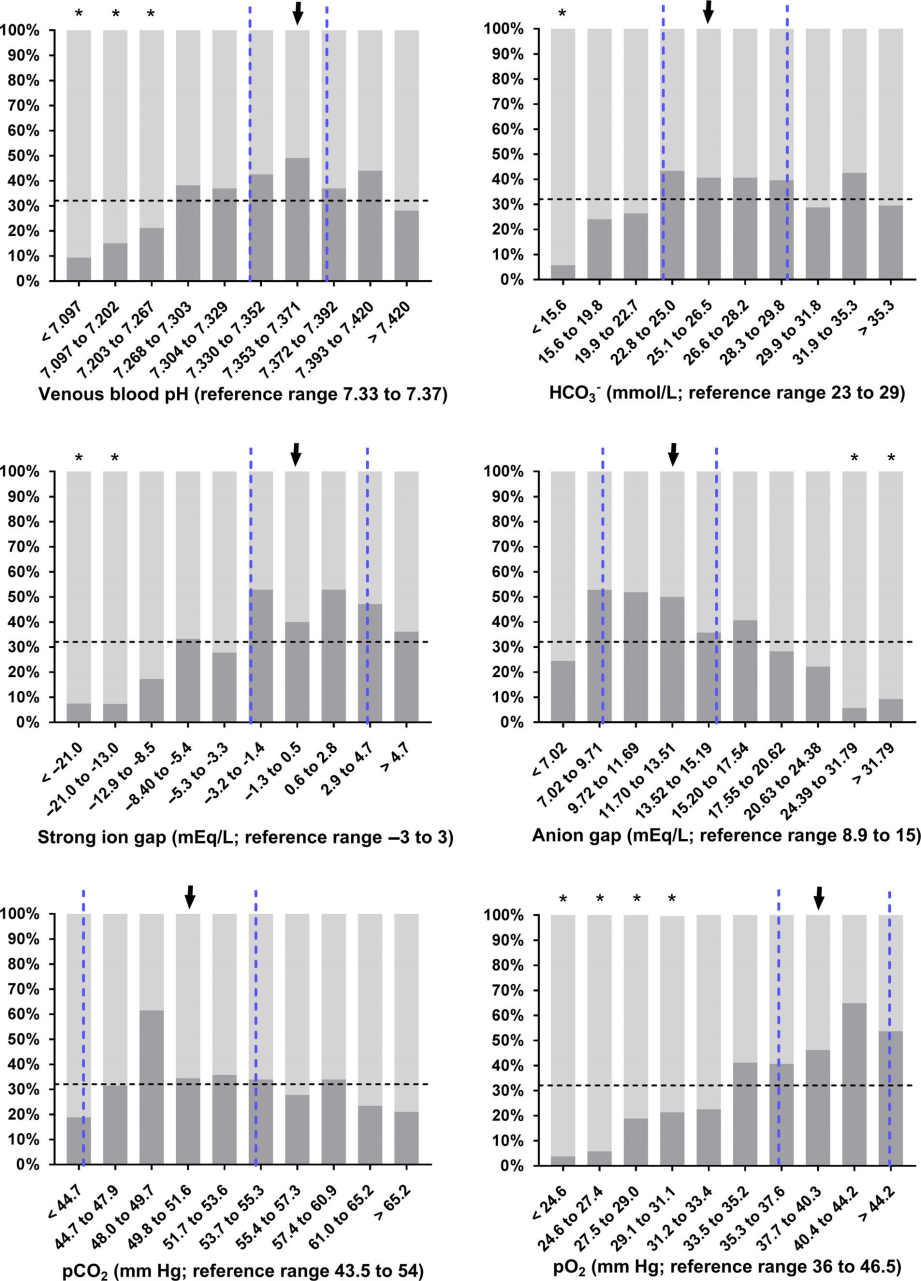
Survival rates (as indicated by dark gray scales) in 535 hospitalized calves with acute surgical abdominal emergencies in deciles of selected blood gas and acid‐base variables. Dashed vertical lines indicate the reference range of respective variables^15^, ^18^ and the dashed horizontal the overall survival rate of calves of this study sample. Survival rates of decile groups that were significantly different (*P* ≤ .006) from the survival rate of the reference group (arrow) are indicated by asterisks

**FIGURE 4:**
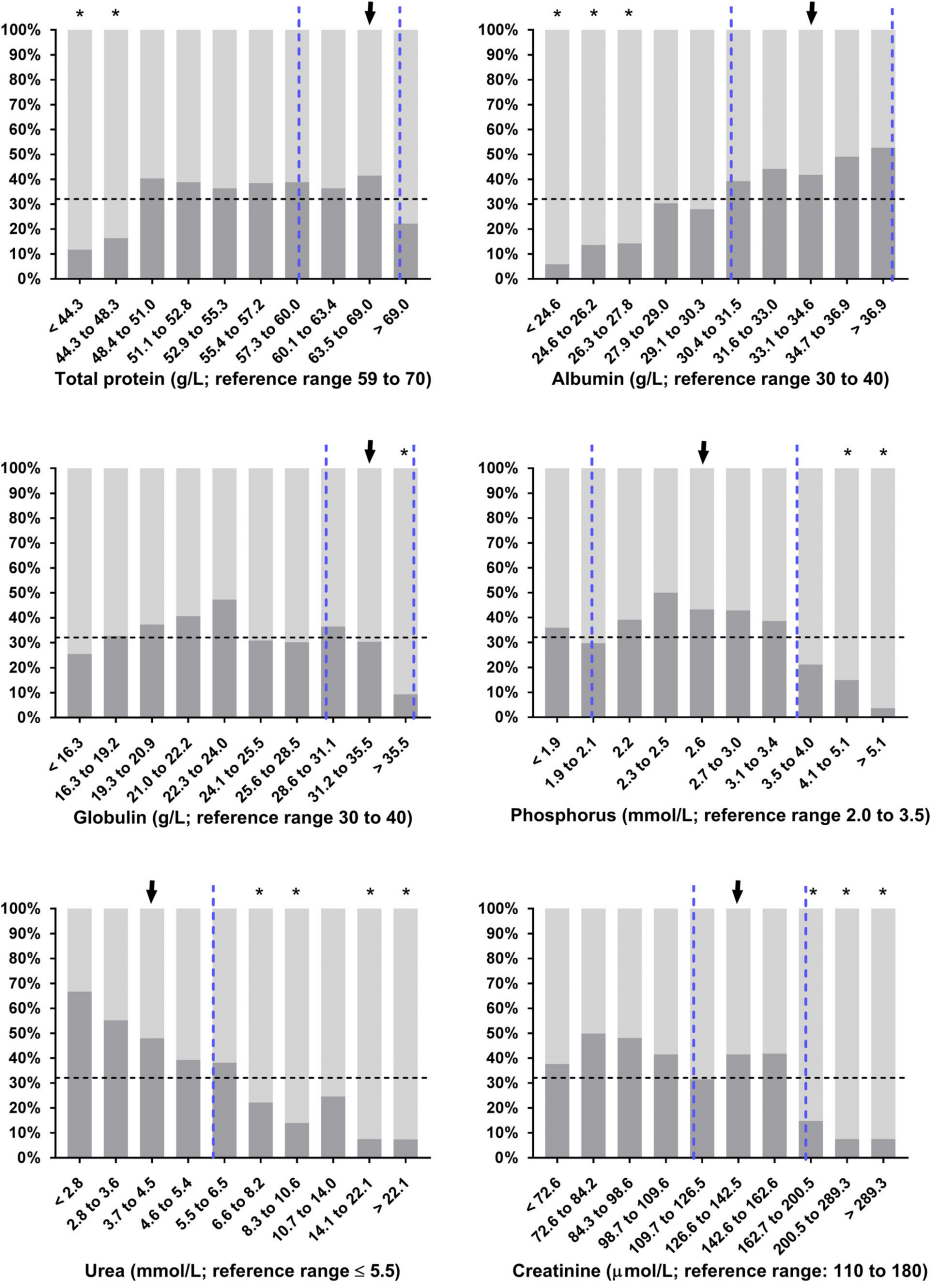
Survival rates (as indicated by dark gray scales) in 535 hospitalized calves with acute surgical abdominal emergencies in deciles of selected biochemical analytes. Dashed vertical lines indicate the reference range of respective variables^18^ and the dashed horizontal the overall survival rate of calves of this study sample. Survival rates of decile groups that were significantly different (*P* ≤ .006) from the survival rate of the reference group (arrow) are indicated by asterisks

**FIGURE 5:**
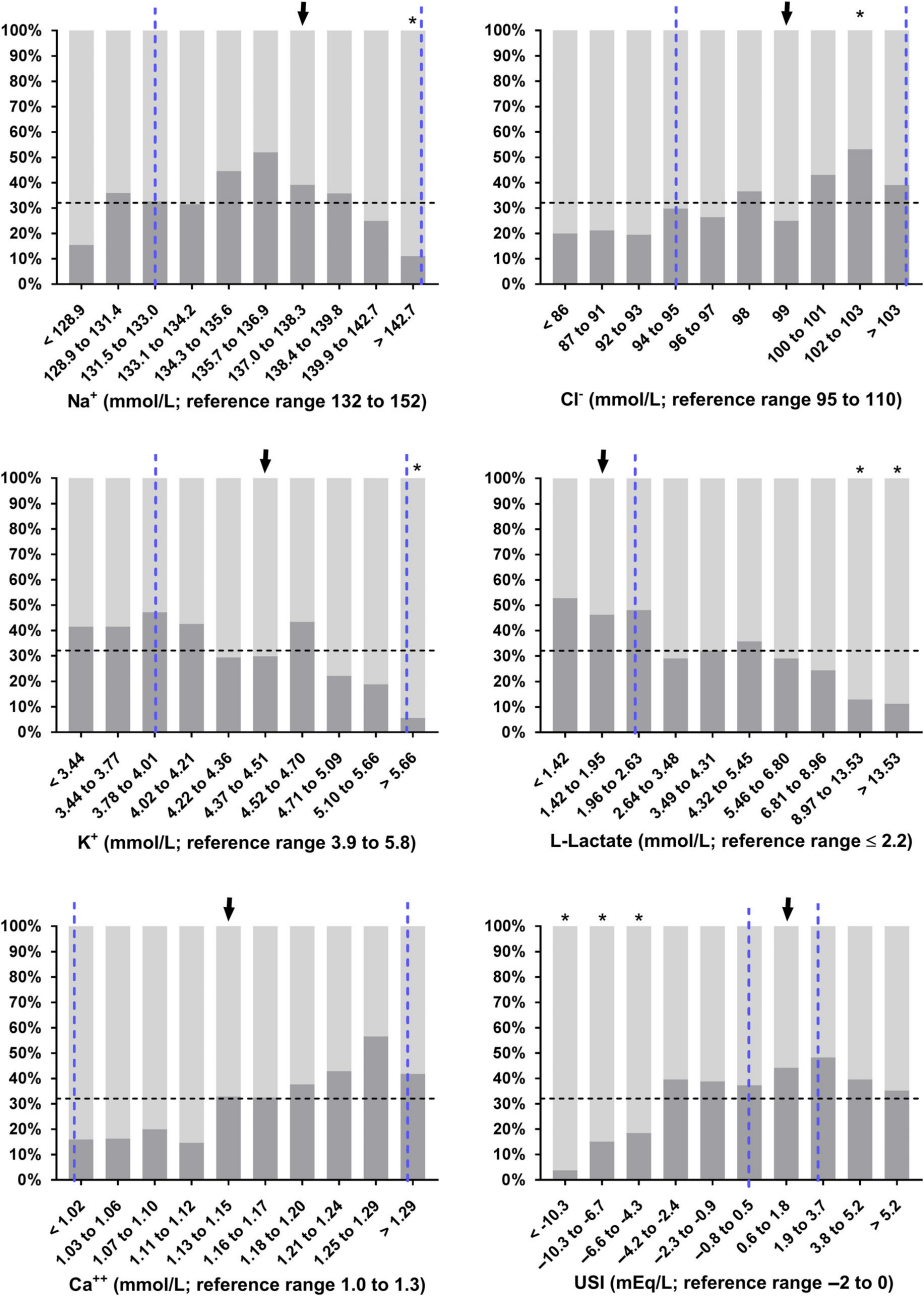
Survival rates (as indicated by dark gray scales) in 535 hospitalized calves with acute surgical abdominal emergencies in deciles of selected electrolytes and biochemical analytes. Dashed vertical lines indicate the reference range of respective variables^17^, ^19^ and the dashed horizontal the overall survival rate of calves of this study population. Survival rates of decile groups that were significantly different (*P* ≤ .006) from the survival rate of the reference group (arrow) are indicated by asterisks

**FIGURE 6:**
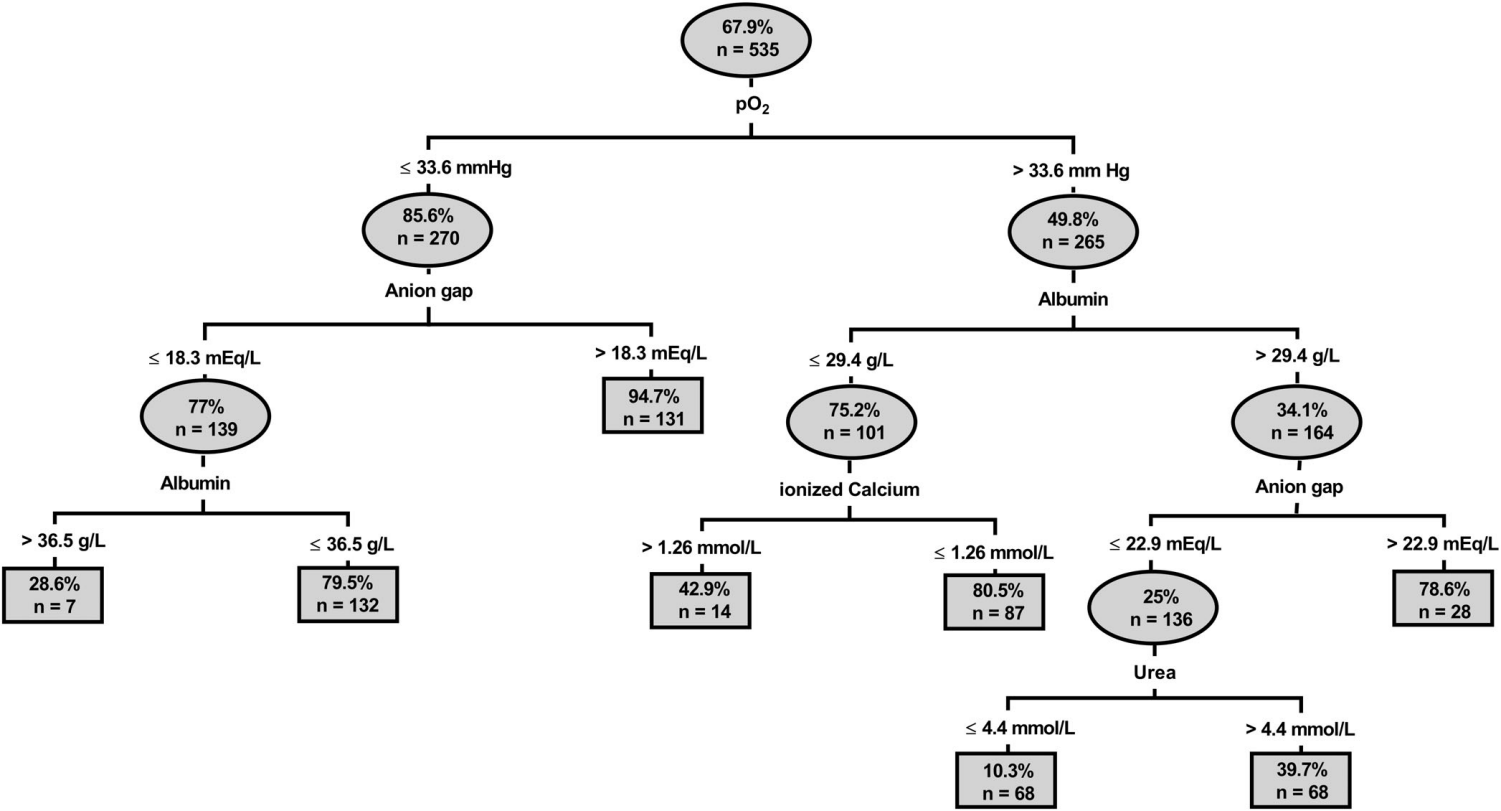
Classification tree illustrating the association between case fatality rate and categories of clinical pathology. Ovals and squares indicate a subset of the population, the number of calves in the subset and the observed mortality rate for this subset. Lines leaving the oval identify a study variable and its cut‐point value that is a significant predictor of death. Branches to the left indicate subgroups with lower case fatality rate, whereas branches to the right indicate subgroups with higher case fatality rate. Subgroups of calves that are not further subdivided by means of a statistically significant predictor of death are indicated by squares. Classification tree analysis indicated that nonsurvival was associated with venous pO_2_ ≤ 33.6 mm Hg. In the 270 calves with venous pO_2_ ≤ 33.6 mm Hg, mortality was further associated with anion gap >18.3 mEq/L, and a serum albumin concentration ≤36.5 g/L in calves with anion gap ≤18.3 mEq/L. In the 265 calves with venous pO_2_ > 33.6 mm Hg, a serum albumin concentration of ≤29.4 g/L was identified as a clinically useful cut‐off value for the prediction of nonsurvival. In calves with serum albumin concentration ≤29.4 g/L, nonsurvival was associated with an ionized calcium concentration ≤1.26 mmol/L. In contrast, in calves with serum albumin concentration >29.4 g/L, nonsurvival was associated with anion gap >22.9 mEq/L, and a serum urea concentration >4.4 mmol/L

Statistically significant differences between survivors and nonsurvivors of DS1 were found for most variables of clinical pathology (Table 5). The proportion of calves with nonsurvival in deciles of selected laboratory variables are shown in Figures 3, 4, 5. Results of the classification tree analysis based on variables as listed in Table 5 are shown in Figure 6. Results of a subsequent multivariable logistic regression analysis with those categories of laboratory variables as independent predictors of nonsurvival are presented in Table 6. The area under the ROC curve of this model was 0.85 (95% CI: 0.82‐0.89, *P* < .001) and the resulting sensitivity, specificity, PPV, and NPV for the prediction of NO at the optimal probability cut‐point of 0.5 was 89.8% (95% CI: 86.3%‐92.5%), 65.7% (95% CI: 58.3%‐72.4%), 84.7% (95% CI: 80.7%‐87.9%), and 75.3% (95% CI: 67.9%‐81.5%), respectively.

**TABLE 5:** Median and interquartile ranges for acid‐base variables, electrolytes, and serum or plasma biochemical analytes in 535 calves with surgical abdominal emergencies stratified by outcome of therapy

	Calves with a positive outcome (n = 172)	Calves with a negative outcome (n = 363)	
Variable	$\tilde{x\;}$ (*Q*_1_/*Q*_3_)	$\tilde{x\;}$ (*Q*_1_/*Q*_3_)	*P*‐value
Henderson‐Hasselbalch acid‐base model			
pH	7.35 (7.30/7.39)	7.31 (7.19/7.38)	<.001
pCO_2_ (mm Hg)	52.5 (48.9/57.3)	54.0 (48.7/60.4)	.07
pO_2_ (mm Hg)	38.4 (34.0/42.2)	30.7 (27.1/36.0)	<.001
HCO_3_^−^ (mmol/L)	27.3 (23.7/30.9)	26.0 (19.9/30.7)	.006
BE(B) (mmol/L)	1.8 (−1.9/5.0)	0.0 (−8.6/4.6)	<.001
AG (mEq/L)	12.7 (9.9/16.3)	17.5 (11.7/25.4)	<.001
SID acid‐base model			
A^−^ (mEq/L)	12.5 (11.1/13.6)	11.4 (9.8/13.0)	<.001
SID_3_ (mEq/L)	40.9 (37.4/45.0)	43.5 (39.1/50.0)	<.001
SID_5_ (mEq/L)	39.5 (36.1/42.5)	39.5 (35.1/44.2)	.81
USI (mEq/L)	0.9 (−2.2/3.4)	−1.8 (−7.3/2.0)	<.001
SIG (mEq/L)	−0.3 (−4.2/3.1)	−5.2 (−13.9/0.4)	<.001
Electrolytes			
Na^+^ (mmol/L)	135.7 (132.9/138.1)	135.7 (132.1/139.9)	.46
K^+^ (mmol/L)	4.17 (3.76/4.55)	4.44 (4.02/5.14)	<.001
Cl^−^ (mmol/L)	99 (95/102)	97 (92/100)	<.001
Ca^2+^ (mmol/L)	1.20 (1.13/1.26)	1.14 (1.08/1.20)	<.001
Ca^2+^_7.40_ (mmol/L)	1.16 (1.09/1.21)	1.07 (1.00/1.14)	<.001
Biochemical analysis			
L‐lactate (mmol/L)	3.1 (1.8/5.4)	5.2 (2.9/9.1)	<.001
Total protein (g/L)	56.1 (51.9/61.5)	54.7 (48.3/61.5)	.05
Albumin (g/L)	32.4 (29.8/35.7)	29.2 (26.2/32.8)	<.001
Globulin (g/L)	23.5 (20.0/28.4)	24.6 (20.1/30.9)	.06
A/G ratio	1.39 (1.15/1.66)	1.17 (0.94/1.5)	<.001
Phosphorus (mmol/L)	2.6 (2.3/3.1)	3.0 (2.3/4.2)	<.001
Urea (mmol/L)	4.5 (3.0/6.4)	8.4 (5.0/15.2)	<.001
Creatinine (μmol/L)	103 (84/138)	140 (99/208)	<.001

*Note*: A negative outcome was defined as death or euthanasia during hospitalization. See legend of Table 2 for explanation of abbreviations.

**TABLE 6:** Multivariable binary logistic regression model for identifying associations between categories of laboratory variables with nonsurvival during hospitalization in 535 calves with acute surgical emergencies

Variable	Coefficient	± SE	OR	95% CI for OR	*P*‐value
Intercept	−3.229	0.51			.04
pO_2_					
≤33.6 mm Hg	1.322	0.246	3.75	2.31‐6.08	<.001
>33.6 mm Hg	Ref.				
Albumin					
≤29.4 g/L	2.747	0.408	15.59	7.01‐34.7	<.001
29.5 to 36.5 g/L	1.198	0.371	3.31	1.60‐6.86	.001
>36.5 g/L	Ref.				
Anion gap					
>22.9 mEq/L	2.554	0.402	12.86	5.85‐28.29	<.001
18.4 to 22.9 mEq/L	1.203	0.362	3.32	1.64‐6.78	<.001
≤18.3 mEq/L	Ref.				
Ionized calcium					
≤1.26 mmol/L	0.695	0.335	2.01	1.04‐3.87	.04
>1.26 mmol/L	Ref.				
Urea					
>4.4 mmol/L	0.908	0.257	2.48	1.50‐4.11	<.001
≤4.4 mmol/L	Ref.				

*Note*: Entered predictors were identified by means of classification tree analysis. Hosmer‐Lemeshow goodness of fit *X* ^2^ = 11.403, df = 7, *P* = .12.

### 3.6 Validation of the prognosis model in calves of DS2

Most of the calves (n = 76; 92%) in DS2 belonged to the German Fleckvieh breed, similar to DS1, and the median age (*Q*_1_/*Q*_3_) was 5.1 (1.4/8.7) weeks. Values for sensitivity, specificity, PPV, and NPV for the prediction of NO until hospital discharge at the probability cut point of 0.5 were 91.5% (95% CI: 81.7%‐96.3%), 62.5% (95% CI: 42.7%‐78.8%), 85.7% (95% CI: 75.0%‐92.3%) and 75.0% (95% CI: 53.1%‐88.8%), respectively. This were similar to those identified using DS1. For the prediction of NO after 3 months after discharge the values for sensitivity, specificity, PPV, and NPV at the probability cut point of 0.5 were 90.5% (95% CI: 80.7%‐95.6%), 70% (95% CI: 48.1%‐85.5%), 90.5% (95% CI: 80.7%‐95.6%), and 70% (95% CI: 48.1%‐85.5%), respectively.

## 4 DISCUSSION

Central findings of this study indicate that acid‐base derangements are common in calves with surgical abdominal emergencies, with acidemia representing a more prevalent disorder than alkalemia. Application of the Henderson‐Hasselbalch acid‐base model indicated that derangements of plasma *c*HCO_3_^−^, increased pCO_2_, and increased concentrations of unmeasured anions (AG) were the most common abnormalities. In contrast application of the simplified strong ion acid‐base model indicated that in addition to increased pCO_2_, acid‐base abnormalities were most commonly characterized by increased concentrations of unmeasured strong anions (SIG), a concomitant decrease of plasma strong ion difference and an increase of plasma nonvolatile weak acids (*A*_tot_). Of relevance is the finding that the simplified strong‐ion acid‐base abnormalities had a higher sensitivity in detecting an acid‐base disorder and therefore more explanatory power than the traditional Henderson‐Hasselbalch approach which is in line to numerous previous investigations.^9^, ^10^, ^11^, ^15^, ^20^

Stepwise linear regression analysis indicated that plasma L‐lactate concentration was the most important predictor of venous blood pH. This was an expected finding based on the high prevalence of hyper‐L‐lactatemia in this study sample^4^, ^12^ that was most likely caused by inadequate oxygen delivery to ischemic gastrointestinal tissues secondary to volvulus, torsion, or luminal distention. The concurrent presence of hyperlactatemia and low venous pO_2_ suggested that global hypoperfusion existed in a number of calves, because of hypovolemic or maldistributive shock secondary to sepsis. However, there is increasing evidence that hyper‐L‐lactatemia can also occur despite apparently adequate oxygen availability. This is especially the case in the presence of sepsis or endotoxemia as a result of a hypermetabolic state, microcirculatory dysfunctions and altered cellular respiration.^21^, ^22^, ^23^ Coefficients of correlation of L‐lactate with venous pO_2_ (*r*_*s*_ = −0.27) and indices of hydration status such as serum urea (*r*_*s*_ = 0.23), creatinine (*r*_*s*_ = 0.51), and phosphorus concentrations (*r*_*s*_ = 0.63) suggest that tissue hypoxia and hypovolemia were contributing factors to hyper‐L‐lactatemia in calves of the present study, but that these were obviously not the single most important mechanisms.

Hypochloremic alkalosis was a rare finding in calves of the present study and hypochloremia was detected in only 35% of calves and was considered mild in most of these cases (Figure 5). This finding is in contrast to previous studies in adult cattle with abomasal volvulus, small intestinal intussusceptions and hemorrhagic bowel syndrome where mean values for plasma or serum chloride concentration between 81 and 91 mmol/L have been reported.^7^, ^24^, ^25^, ^26^ This indicates that sequestration of chloride in the abomasum or cranial part of the small intestine as observed in experimental settings,^27^ occurs to a greater extent in adult cattle with gastrointestinal ileus than in affected calves, as reported in a previous study.^28^ For those reasons, we hypothesize that chloride is a much better predictor of venous blood pH in adult cattle with acute abdominal emergencies than in calves of the present study sample as hypochloremia increases plasma strong ion difference and therefore favors the occurrence of strong ion (metabolic) alkalosis.^29^ A possible explanation for the low prevalence of marked hypochloremia in the study sample of critically ill calves with abdominal disorders might be that many acute abdominal disease conditions in calves are characterized by a more rapid disease progression than in adult cattle.^4^ This has been especially documented for calves with abomasal tympany or volvulus, where extreme distension of the abomasum with gas and fluid can rapidly lead to irreversible organ damage and shock within a few hours.^6^, ^30^ Another plausible explanation for a more rapid disease progression in calves is the lower degree of fat deposition in the omentum and mesentery when compared to adult animals.^4^ This makes calves with acute abdominal emergencies potentially more vulnerable to ischemic or necrotizing lesions and therefore an acidemic state because of a rapid increase of plasma L‐lactate and other unmeasured strong anions resulting in a strong ion acidosis (Figures 1 and 2).

Classification tree analysis indicated that venous oxygen tension was the most important clinicopathologic predictor of death in this study sample. A venous pO_2_ of 33.6 mm Hg was identified as the best clinicopathologic variable that categorized calves in 2 groups that differed markedly in their survival rate (14.4% vs 50.2%; Figure 6). Similar findings were obtained in a recent study of calves with naturally acquired sepsis, in that a venous pO_2_ of >22.9 mm Hg was a good predictor of survival (Se = 0.9; Sp = 0.9).^31^ The oxygen tension in venous blood depends on a variety of factors including the extent of oxygenation of arterial blood, the blood flow to the tissues, the metabolic rate of the tissue drained by the veins from which blood is sampled, and the transit time of blood through capillaries.^17^ Acute and sustained decreases in arterial and mixed venous pO_2_ and buccal mucosal oxygen saturation occur in calves with experimentally‐induced endotoxemia^32^, ^33^ and naturally acquired sepsis.^34^ The exact reasons for decreased venous pO_2_ often remains unknown in clinical situations but it generally indicates inadequate oxygen delivery to tissues resulting in an increased oxygen extraction ratio.^17^

Inadequate O_2_ delivery or increased O_2_ consumption in critically ill calves, if sufficiently severe in magnitude or prolonged in duration, results in an oxygen deficit and increasing oxygen debt that is most easily detected in a clinical setting by measuring venous blood pO_2_ and L‐lactate concentration. Concurrent monitoring of venous blood pO_2_ and L‐lactate concentration can help the clinician recognize the different stages of shock, in that normal venous blood pO_2_ and L‐lactate concentrations indicate adequate O_2_ delivery. In comparison, a low venous pO_2_ accompanied by normal L‐lactate concentrations indicates increased tissue oxygen extraction and a developing oxygen deficit, whereas the concurrent presence of low venous blood pO_2_ and hyperlactatemia, as in the calves in this study, indicates the presence of an oxygen debt because of anerobic cellular metabolism.

The combination of increased AG and low venous oxygen tension resulted in a high PPV for a negative outcome of therapy of 94.7% in a subset of 131 calves. This finding is consistent with the results of a study in critically ill neonatal foals where AG and venous oxygen tension were also identified as predictors of death.^35^ Furthermore, AG is associated with a negative outcome of therapy in cows with right‐sided displacement of the abomasum or abomasal volvulus,^24^, ^36^ although this was not consistently observed.^25^, ^37^ In this context, a further interesting finding of this study, was that AG (representing the unmeasured anion concentration) appeared to be a more reliable predictor of death in the multivariable classification tree analysis than plasma L‐lactate concentration or even SIG (representing the unmeasured strong anion concentration). One possible explanation for this finding was that increased serum inorganic phosphorus concentration was additionally identified as a prognostic factor in this study sample which was, aside from L‐lactate, an important predictor of increased AG (Figure 2). Based on previous findings in cows with right‐sided abomasal displacement^38^ and calves with neonatal diarrhea,^9^, ^39^ hyperphosphatemia was likely the result of dehydration and decreased renal perfusion, although tissue hypoxia could have been another contributing factor.^40^, ^41^ Furthermore, negative values for the calculated USI concentrations indicated the presence of unidentified strong anions in many calves which also contributed to an increased AG. The nature of these anions remains speculative, but it is conceivable that they are partially uremic anions such as sulfate that accumulated because of dehydration and decreased glomerular filtration rate. Also, metabolites such as pyruvate, as observed in cows with abomasal volvulus,^42^ or ß‐hydroxybutyrate might have contributed to the USI concentration. Unidentified strong anions are a well‐known feature of critically ill animals and humans^11^, ^13^, ^43^, ^44^, ^45^ and some studies have documented an association between increased USI concentrations and the presence of endotoxemia, sepsis, and inflammatory cytokines.^46^, ^47^, ^48^ Taken together, the associations of AG to L‐lactate, phosphorus, USI, and pH, likely makes AG a global indicator of tissue hypoxia, acidemia, and hydration status and therefore a clinically useful predictor of a negative postoperative outcome in this multivariable approach. Higher serum concentrations of urea and creatinine concentrations in calves with a NO further indicated that a reduced hydration status and a concomitant decrease in glomerular filtration rate is a negative prognostic factor in calves undergoing abdominal surgery. Based on the results of the performed uni‐ and multivariable analyses (Figures 4 and 6), it appears that serum urea concentration had more explanatory power than serum creatinine concentration. This might be related to urea being a more sensitive indicator of reduced urine flow rate, as it is dependent both on glomerular filtration and tubular reabsorption, whereas creatinine clearance exclusively depends on glomerular filtration rate.^49^

Another condition that was significantly associated with a negative outcome was hypoalbuminemia. This finding is in line with studies in critically ill humans that underwent surgery to correct an acute abdominal problem.^50^, ^51^ An obvious explanation for the association between hypalbuminemia and NO in calves of the present study was leakage of albumin in the abdominal cavity because of intraabdominal inflammation. Peritonitis in dairy cows is associated with increased albumin concentrations in peritoneal fluid and consequently an increased albumin peritoneal‐fluid‐to‐blood‐ratio and a decreased serum‐ascites albumin gradient.^52^ In the present study, no clear association between hypoalbuminemia and intraoperative diagnosis of peritonitis was detected (Table 2). Nevertheless, albumin is also known as a negative acute phase protein, and hypoalbuminemia could therefore additionally be related to a systemic inflammatory response because of endotoxemia or sepsis and therefore a higher risk for a NO.^53^, ^54^, ^55^, ^56^

Although this study provided valuable information in respect to derangement of acid‐base variables and associated biochemical analytes, there were some limitations that were also considered relevant in our previous analyses.^3^, ^4^, ^12^ These limitations include the selection bias of cases that are referred to veterinary teaching hospitals, the potential impact of euthanasia on the outcome, interindividual treatment variations, and documentation issues related to the retrospective part of the study. As there are age‐dependent changes on acid‐base and electrolyte variables,^57^ the use of age‐invariant reference ranges might be considered as another limitation. A further major limitation was that the established prognosis model required comprehensive and expensive laboratory equipment, which markedly limits its applicability in ambulatory field practice. This issue is considered a major difference to previous prognosis models that were based on L‐lactate and glucose,^4^, ^12^ as these can be easily applied by use of portable L‐lactate and glucose analyzers, but that were characterized by lower predictive accuracies. However, recent years have seen a major progress with regards to availability and validation of animal‐side diagnostic tests in bovine medicine.^58^, ^59^ Portable devices also allow for calf‐side blood‐gas and electrolyte analysis^60^ and it is likely that more of these analyzers with a broad diagnostic spectrum will become available at lower cost in the future.

## 5 CONCLUSIONS

Surgical abdominal emergencies in calves frequently result in complex metabolic derangements including acid‐base disorders that are associated with disease severity and consequently increased mortality risk. Findings of the present study indicated that low venous oxygen tension, hyper L‐lactatemia, high anion gap, azotemia, and hypoalbuminemia have clinical utility for the prediction of a negative outcome of therapy in affected calves.

## CONFLICT OF INTEREST DECLARATION

Authors declare no conflict of interest.

## OFF‐LABEL ANTIMICROBIAL DECLARATION

Authors declare no off‐label use of antimicrobials.

## INSTITUTIONAL ANIMAL CARE AND USE COMMITTEE (IACUC) OR OTHER APPROVAL DECLARATION

The prospective part of this study was approved by the ethics committee of the Centre of Veterinary Clinical Medicine, LMU Munich (permit no. 39‐15‐01‐2015). For the retrospective part of the study, authors declare no IACUC or other approval was needed.

## HUMAN ETHICS APPROVAL DECLARATION

Authors declare human ethics approval was not needed for this study.

## Supporting information


**Table S1**. Spearman's coefficients of correlation between selected clinicopathologic findings in 535 calves with acute abdominal emergencies.
