# Supplementary material for: Acid‐base imbalances and the association of blood‐gas variables, electrolytes, and biochemical analytes with outcome in hospitalized calves undergoing abdominal surgery
Source: J Vet Intern Med. 2023 Jan 20;37(2):740–56. doi: 10.1111/jvim.16618 (PMC10061191; doi:10.1111/jvim.16618)
Supplement: Supplementary file 1 — Table S1. Spearman's coefficients of correlation between selected clinicopathologic findings in 535 calves with acute abdominal emergencies. [file JVIM-37-740-s001.pdf]

Supplemental Table S1. Spearman's coefficients of correlation between selected clinicopathologic findings in 535 calves with acute abdominal emergencies.

	**pH**	**pCO_2**	**pO_2**	**HCO_3^-**	**BE**	**SID_3**	**SID_5**	**SID_{eff}**	**USI**	**AG**	**SIG**	**Na^+**	**K^+**	**Cl^-**	**Ca^{2+}**	**L-lac**	**TP**	**Alb**	**P**	**Urea**	**Crea**
**pH**	1.0.																				
**pCO_2**	-0.07^{NS}	1.0																			
**pO_2**	0.20^{**}	-0.36^{**}																			
**HCO_3^-**	0.83^{**}	0.42^{**}	-0.03^{NS}	1.0																	
**BE**	0.91^{**}	0.28^{**}	0.04^{NS}	0.98^{**}	1.0																
**SID_3**	-0.06^{NS}	0.47^{**}	-0.36^{**}	0.18^{**}	0.10^{*}	1.0															
**SID_5**	0.42^{**}	0.42^{**}	-0.17^{**}	0.62^{**}	0.57^{**}	0.68^{**}	1.0														
**SID_{eff}**	0.82^{**}	0.36^{**}	0.0^{NS}	0.97^{**}	0.95^{**}	0.23^{**}	0.66^{**}	1.0													
**USI**	0.68^{**}	0.03^{NS}	0.23^{**}	0.63^{**}	0.68^{**}	-0.41^{**}	-0.08^{NS}	0.63^{**}	1.0												
**AG**	-0.67^{**}	0.05^{NS}	-0.27^{**}	-0.59^{**}	-0.64^{**}	0.63^{**}	0.10^{*}	-0.51^{**}	-0.84^{**}	1.0											
**SIG**	0.74^{**}	-0.04^{NS}	0.28^{**}	0.66^{**}	0.71^{**}	-0.53^{**}	0.02^{NS}	0.64^{**}	0.90^{**}	-0.96^{**}	1.0										
**Na^+**	-0.19^{**}	0.30^{**}	-0.12^{*}	-0.04^{NS}	-0.09^{*}	0.51^{**}	0.28^{**}	-0.03^{**}	-0.38^{**}	0.42^{**}	-0.39^{**}	1.0									
**K^+**	-0.50^{**}	0.04^{**}	-0.30^{**}	-0.42^{**}	-0.46^{**}	0.11^{*}	-0.11^{*}	-0.44^{**}	-0.52^{**}	0.42^{**}	-0.48^{**}	0.08^{NS}	1.0								
**Cl^-**	-0.17^{**}	-0.25^{**}	0.27^{**}	-0.29^{**}	-0.26^{**}	-0.63^{**}	-0.50^{**}	-0.33^{**}	0.05^{NS}	-0.28^{**}	0.19^{**}	0.26^{**}	0.04^{NS}	1.0							
**Ca^{2+}**	-0.31^{**}	-0.02^{NS}	0.19^{**}	-0.29^{**}	-0.30^{**}	-0.23^{**}	-0.22^{**}	-0.28^{**}	-0.16^{**}	0.02^{NS}	-0.05^{NS}	0.12^{*}	0.16^{**}	0.39^{**}	1.0						
**L-Lac**	-0.59^{**}	0.16^{**}	-0.27^{**}	-0.47^{**}	-0.52^{**}	0.53^{**}	-0.14^{*}	-0.43^{**}	-0.47^{**}	0.79^{**}	-0.78^{**}	0.29^{**}	0.27^{**}	-0.29^{**}	0.10^{*}	1.0					
**TP**	-0.09^{*}	0.07^{NS}	0.0^{NS}	-0.04^{NS}	-0.07^{NS}	0.37^{**}	0.29^{**}	0.17^{**}	-0.05^{NS}	0.34^{**}	-0.12^{*}	0.15^{*}	-0.01^{NS}	-0.25^{**}	0.03^{NS}	0.21^{**}	1.0				
**Alb**	-0.14^{*}	0.07^{NS}	0.13^{*}	-0.10^{*}	-0.12^{*}	0.27^{**}	0.15^{*}	0.02^{NS}	-0.12^{*}	0.29^{**}	-0.17^{**}	0.14^{*}	-0.08^{NS}	-0.17^{**}	0.13^{**}	0.21^{**}	0.58^{**}	1.0			
**P**	-0.52^{**}	0.23^{**}	-0.33^{**}	-0.37^{**}	-0.44^{**}	0.59^{**}	0.14^{*}	-0.33^{**}	-0.61^{**}	0.76^{**}	-0.71^{**}	0.32^{**}	0.44^{**}	-0.31^{**}	-0.14^{**}	0.63^{**}	0.31^{**}	0.29^{**}	1.0		
**Urea**	-0.03^{NS}	0.03^{NS}	-0.35^{**}	0.0	-0.01^{NS}	0.35^{**}	0.27^{**}	0.03^{NS}	-0.22^{**}	0.31^{**}	-0.27^{**}	-0.10^{*}	0.08^{NS}	-0.47^{**}	-0.55^{**}	0.23^{**}	0.15^{*}	-0.09^{*}	0.41^{**}	1.0	
**Crea**	-0.28^{**}	0.15^{*}	-0.30^{**}	-0.19^{**}	-0.23^{**}	0.53^{**}	0.21^{**}	-0.15^{**}	-0.45^{**}	0.59^{**}	-0.54^{**}	0.22^{**}	0.33^{**}	-0.36^{**}	-0.31^{**}	0.51^{**}	0.22^{**}	0.14^{*}	0.65^{**}	0.59^{**}	1.0

** $P < 0.001$, * $P < 0.05$, ^{NS} not significant

pCO_2 = partial pressure of carbon dioxide, BE = base excess, AG = anion gap. A_{tot} = concentration of non-volatile weak acids, SID_3 = strong ion difference calculated from three strong ions, SID_5 = strong ion difference calculated from five strong ions, SID_{eff} = effective strong ion difference, USI = unidentified strong ions, SIG = strong ion gap, L-Lac = L-lactate, TP = total protein, Alb = albumin, Crea = creatinine
